# Supplementary material for: Neuroprotective and Neurotrophic Potential of Flammulina velutipes Extracts in Primary Hippocampal Neuronal Culture
Source: Nutrients. 2025 Sep 30;17(19):3107. doi: 10.3390/nu17193107 (PMC12526165; doi:10.3390/nu17193107)
Supplement: Supplementary file 1 [file nutrients-17-03107-s001.zip › nutrients-3820909-supplementary.pdf]

Table S1. List of compounds identified from GC-MS analysis (ethanol extract, FVEE) along with their retention time, peak area, and 2D structure.

Serial No.	Compound Name	RT (min)	Peak Area (%)	Canonical Smiles	2D Structure
1	DIMETHYL SULFOXIDE	5.883	96.46875	CS(=O)C	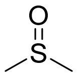
2	DIMETHYL SULFONE	8.304	0.061935	CS(=O)(=O)C	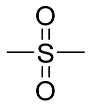
3	LINALOOL	9.169	0.089509	CC(=CCCC(C)(C=C)O)C	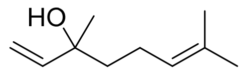
4	DODECANE, 1-FLUORO-	9.712	0.020922	CCCCCCCCCCCCF	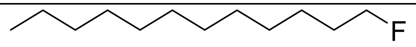
5	HENTRIACONTANE	14.774	0.02557	CCCCCCCCCCCCCCCCCCCCCCCCCCCCCCC	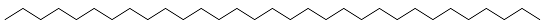
6	SILANE, DIMETHYL(3-METHYLBUT-3-ENYLOXY)PROPOXY-	20.164	0.052185	CCCO[Si](C)(C)OCCC(=C)C	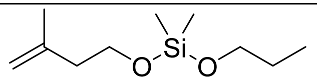
7	2-PHENYLCYCLOHEXANONE	20.862	0.177734	C1CCC(=O)C(C1)C2=CC=CC=C2	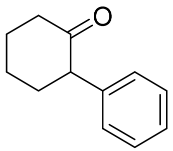
8	2-PHENYLCYCLOHEXANONE	21.458	0.034971	C1CCC(=O)C(C1)C2=CC=CC=C2	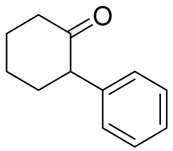

9	5-ACETOXYMETHYL-2,6,10-TRIMETHYL-2,9-UNDECADIEN-6-OL	30.247	0.113173	CC(=CCCC(C)(C(CC=C(C)C)COC(=O)C)O)C	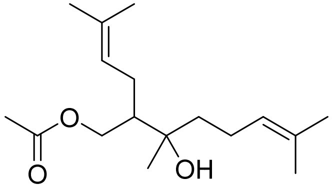
10	6,9-OCTADECADIENOIC ACID, METHYL ESTER	32.646	0.319596	CCCCCCCC/C=C/C/C=C/CCCCC(=O)OC	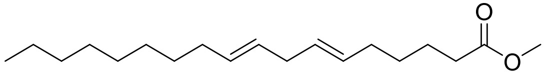
11	1H-INDOLE, 2,3-DIHYDRO-1-METHYL-	34.369	0.232693	CN1CCC2=CC=CC=C21	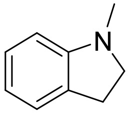
12	SILANE, DIMETHYL(DIMETHYL(BUT-3-ENYLOXY)SILYLOXY)ISOBUTOXY-	38.15	0.64714	CC(C)CO[Si](C)(C)O[Si](C)(C)OCCC=C	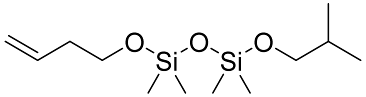
13	5(4H)-ISOXAZOLONE, 4,4'-AZOBIS[3-PHENYL-	14.548	0.351277	C1=CC=C(C=C1)C2=NOC(=O)C2N=NC3C(=NOC3=O)C4=CC=CC=C4	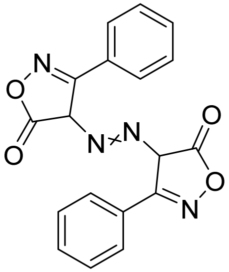
14	QUINAZOLINE, 7-NITRO-	16.948	0.589554	C1=CC2=CN=CN=C2C=C1[N+](=O)[O-]	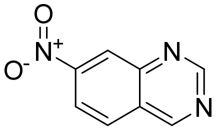

15	DIGLYCOLIC ACID, 2,4-DICHLORO-6-FORMYLPHENYL ETHYL ESTER	18.229	0.566151	CCOC(=O)COCC(=O)OC1=C(C=C(C=C1Cl)Cl)C=O	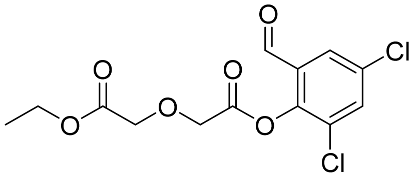
16	AZETIDINE, 3-METHYL-3-PHENYL-	20.871	0.098694	CC1(CNC1)C2=CC=CC=C2	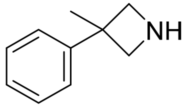
17	1-HYDROXYINDEN-3-ONE-1-CARBOXYLIC ACID	21.629	1.184387	C1C(=O)C2=CC=CC=C2C1(C(=O)O)O	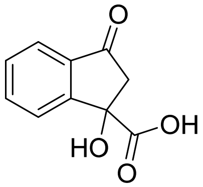
18	PHTHALIC ACID, 4-BROMOPHENYL ETHYL ESTER	24.681	1.244677	CCOC(=O)C1=CC=CC=C1C(=O)OC2=CC=C(C=C2)Br	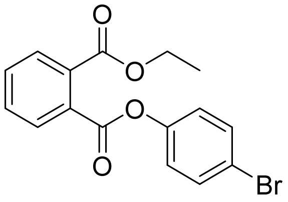
19	RIBITOL	25.774	0.246698	C([C@H](O)[C@H](O)[C@H](O)CO)O	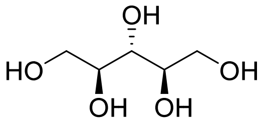
20	(2R,3R,6S)-6-ISOPROPYL-3-METHYL-2-(PROP-1-EN-2-YL)-3-VINYLCYCLOHEXANON	26.874	0.516601	CC(C)C1CCC(C(C1=O)C(=C)C)(C)C=C	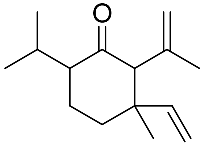
21	DECANOIC ACID, 2-METHYL-	28.337	0.61588	CCCCCCCCC(C)C(=O)O	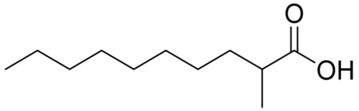

Table S2. List of compounds identified from GC-MS analysis (methanol extract, FVME) along with their retention time, peak area, and 2D structure.

Serial No.	Compound Name	RT (min)	Peak Area (%)	Canonical Smiles	2D Structure
1	BENZENE, 1,3-DIMETHYL-	4.288	15.6289	CC1=CC(=CC=C1)C	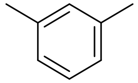
2	BENZENE, 1,3-DIMETHYL-	4.844	3.519971	CC1=CC(=CC=C1)C	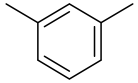
3	DIMETHYLSULFOXONIUM FORMYLMETHYLIDE	5.73	64.90911	CS(=CC=O)(=O)C	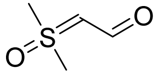
4	LINALOOL	9.162	0.233511	CC(=CCCC(C)(C=C)O)C	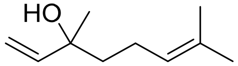
5	TRANS-2,4-DIMETHYLTHIANE, S,S-DIOXIDE	9.846	0.03455	CC1CCS(=O)(=O)C(C1)C	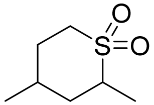
6	ALPHA.-METHYLTYROSINE, N,O-BIS(PENTAFLUOROPROPIONYL)-	26.456	0.023354	CC(CC1=CC=C(C=C1)OC(=O)C(C(F)(F)F)(F)F)(C(=O)O)NC(=O)C(C(F)(F)F)(F)F	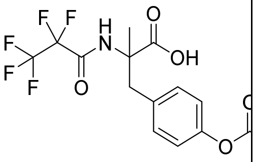
7	METHYL 11-METHYL-DODECANOATE	27.992	0.026906	CC(C)CCCCCCCCCC(=O)OC	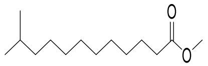
8	9-OCTADECENAMIDE, (Z)-	34.911	4.158982	CCCCCCCC/C=C\CCCCCCCC(=O)N	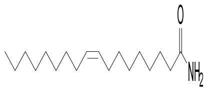
9	DODECANE, 1-FLUORO-	7.494	0.38183	CCCCCCCCCCCCF	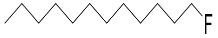

10	TRICYCLO[3.3.0.0(2,8)] OCTAN-3-ONE, 4-[2-(M-ANISYL)ETHYL]-8-METHYL-	8.534	0.21857	CC12CCC3C1C2C(=O)C3CCCC4=CC(=CC=C4)OC	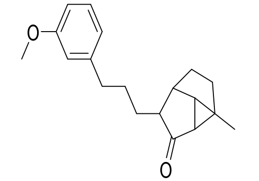
11	METHYL 3,3-DIMETHOXYPROPIONATE	18.398	0.89704	COC(CC(=O)OC)OC	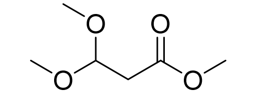
12	3-CYCLOPENTYLPROPIONAMIDE, N-METHALLYL-	21.886	0.49384	CC(=C)CNC(=O)CCC1CCCC1	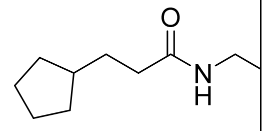
13	DIETHYLMALONIC ACID, MONOCHLORIDE, TETRAHYDROFURFURYL ESTER	22.013	0.62597	CCC(CC)(C(=O)OCC1CCCO1)C(=O)Cl	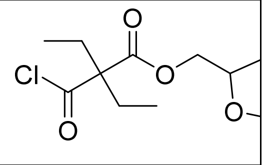
14	DIETHYL PHTHALATE	24.675	0.79378	CCOC(=O)C1=CC=CC=C1C(=O)OCC	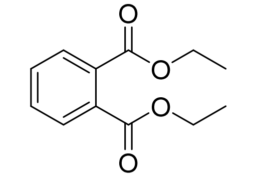
15	2-CYCLOPENTEN-1-ONE, 2-(2-BUTENYL)-3-METHYL-, (Z)-	26.928	0.80061	C/C=C\CC1=C(CCC1=O)C	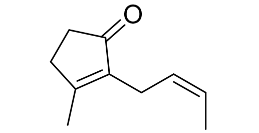
16	NONANOIC ACID, METHYL ESTER	28.344	0.12300	CCCCCCCCC(=O)OC	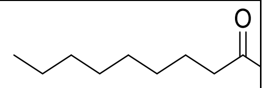
17	BENZENEMETHANOL, .ALPHA.-(2-AMINOCYCLOPENTYL)-	29.765	1.69832	C1CC(C(C1)N)C(C2=CC=CC=C2)O	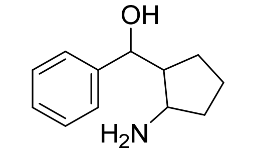

18	2,4-DODECADIENOIC ACID, 11-METHOXY-3,7,11-TRIMETHYL-, METHYL ESTER, (E,E	29.9 19	0.075 78	CC(CCCC(C)(C)OC)C/C=C/C(=C/C(=O)OC)/C	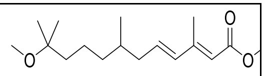
19	METHYL 2,6-ANHYDRO-.ALPHA.-D-ALTROSIDE	30.1	0.162 29	COC1C2C(C(C(O1)CO2)O)O	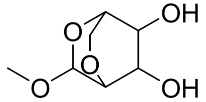
20	3-METHYL-2-(2-OXOPROPYL)FURAN	30.2 81	0.122 30	CC1=C(OC=C1)CC(=O)C	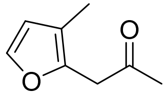
21	ADIPIC ACID, DICYCLOBUTYL ESTER	36.8 33	62.62 398	C1CC(C1)OC(=O)CCCCC(=O)OC2CCC2	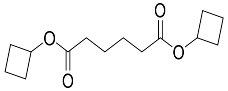
22	HEPTANAMIDE, 4-ETHYL-5-METHYL-	38.6 64	0.229 34	CCC(C)C(CC)CCC(=O)N	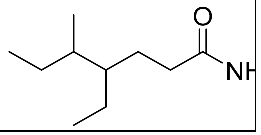

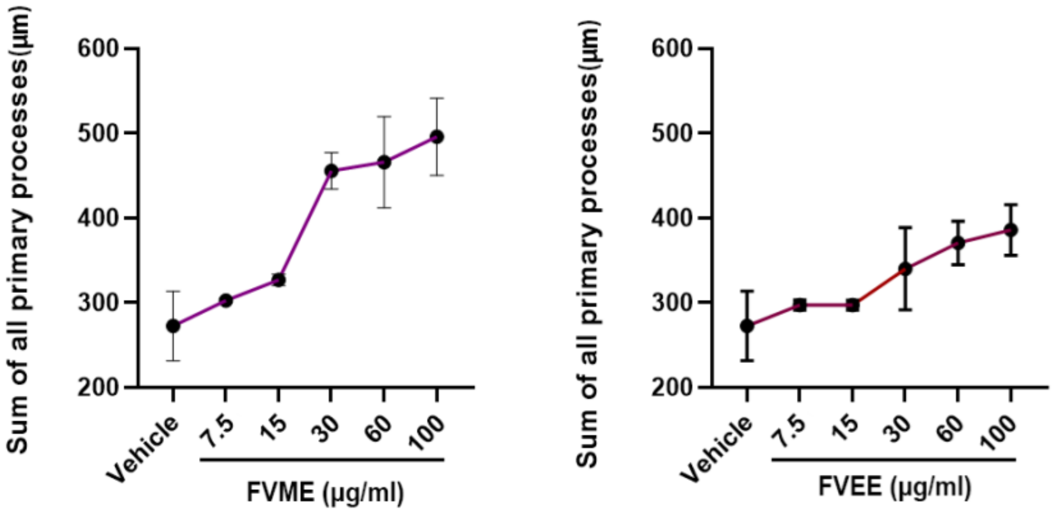

Figure S1. Dose-dependent effect of FVME and FVEE on neuritogenesis in primary hippocampal neurons. The line plot represents the average number of primary processes per neuron. Data are presented as mean ± SD from three independent biological replicates. FVME, *Flammulina velutipes* methanolic extract; FVEE, *Flammulina velutipes* ethanolic extract.

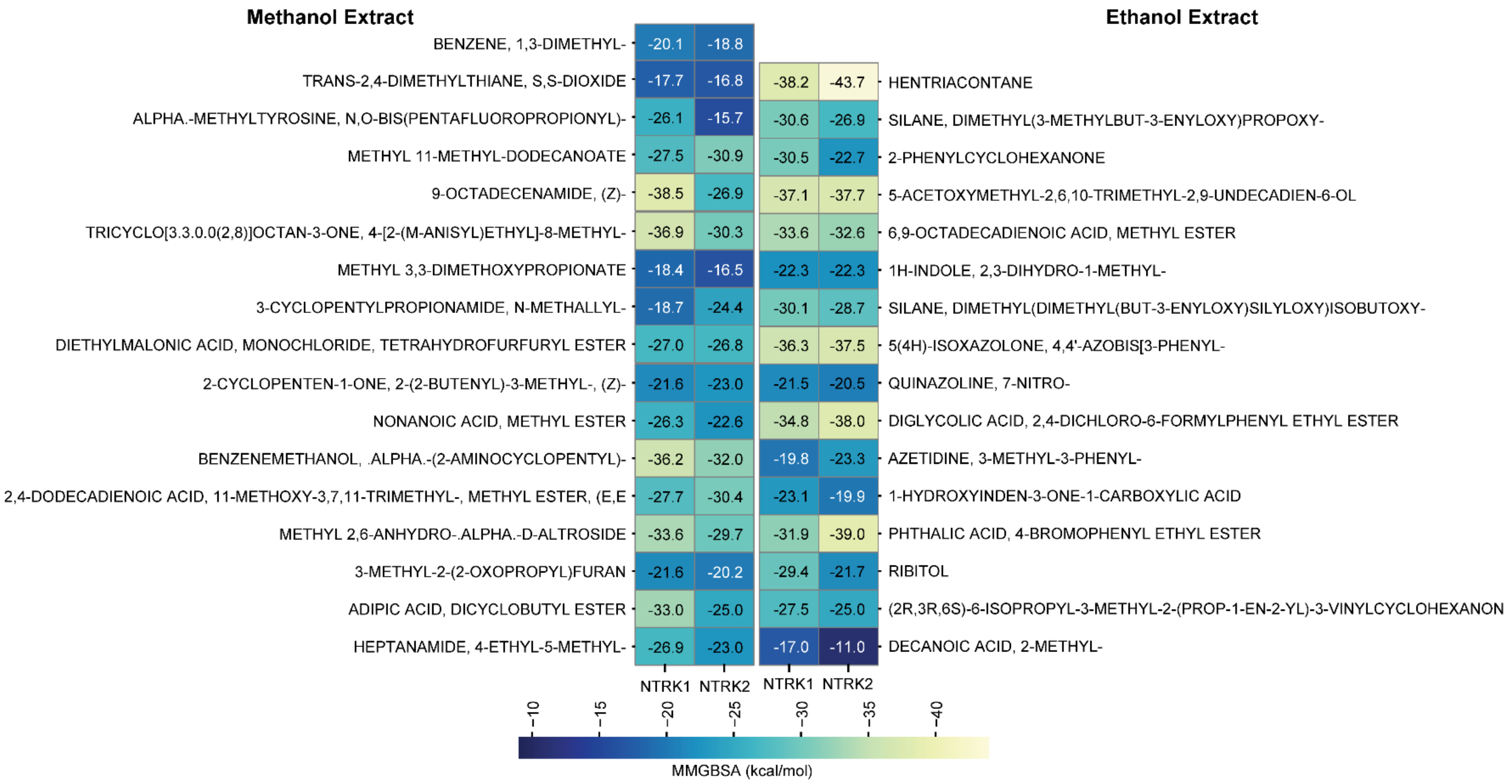

Figure S2. Color-coded Heatmap illustrating the MMGBSA binding energy of compounds found in methanol (left panel, FVME) and ethanol (right panel, FVEE) extracts of *Flammulina velutipes*.

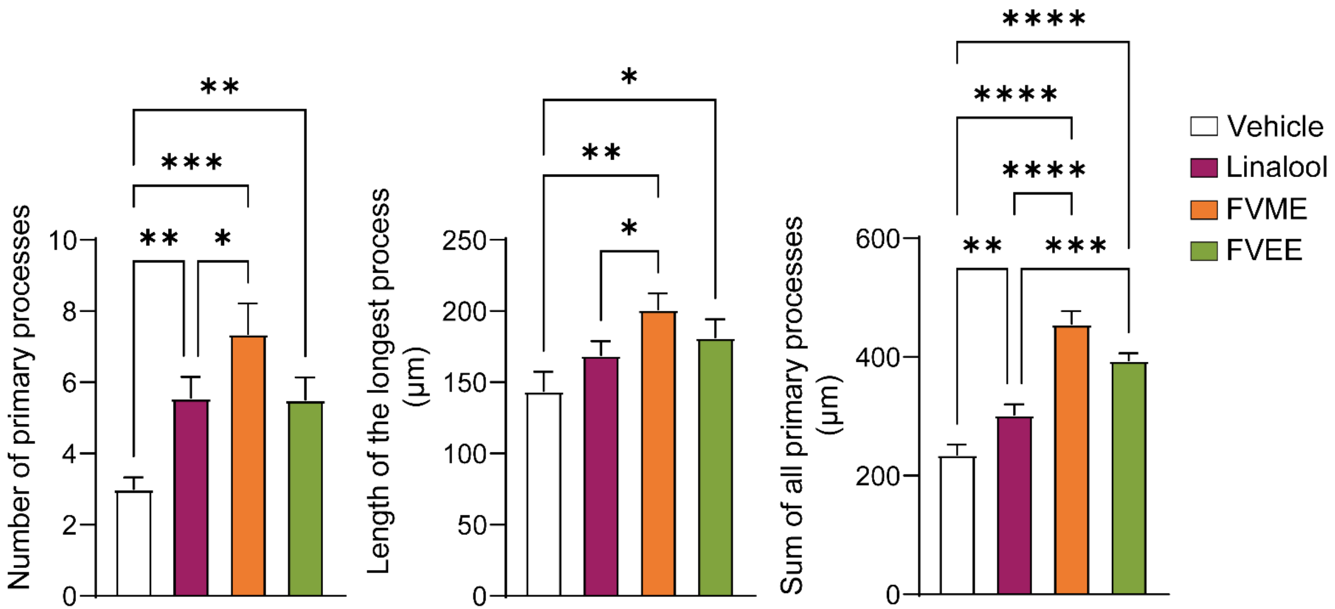

Figure S3: Neuritogenic effect of Linalool on primary neuronal culture. Morphological analysis showing the number of primary processes, the length of the longest process, and the total length of the primary processes. Data are presented as mean ± SD from three independent biological replicates. Statistical significance was assessed by one-way ANOVA followed by Dunnett's multiple comparisons test versus vehicle (* $p < 0.05$, ** $p < 0.01$, *** $p < 0.001$, **** $p < 0.0001$). FVME, *Flammulina velutipes* methanolic extract; FVEE, *Flammulina velutipes* ethanolic extract.
